# Loss of *eif-2alpha* phosphorylation on S49 (mammalian S51) associated with the integrated stress response hastens development in *C. elegans​*

**DOI:** 10.17912/W2BM1S

**Published:** 2017-11-22

**Authors:** Jarod Rollins, Noah Lind, Aric N. Rogers

**Affiliations:** 1 Mount Desert Island Biological Laboratory,159 Old Bar Harbor Rd Salisbury Cove, ME 04672, United States of America.

**Figure 1.**
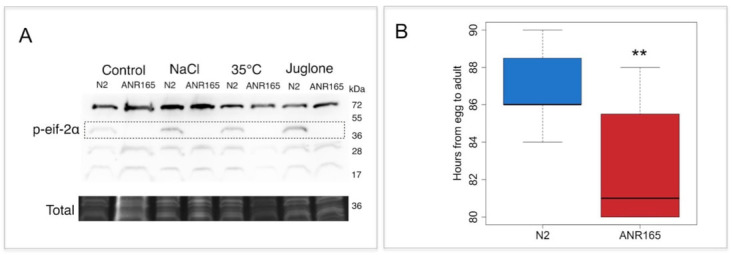


## Description

The gene *eif-2alpha* (Y37E3.10) in *C. elegans* encodes for the alpha subunit of eukaryotic translation initiation factor 2 (eIF2). The eIF2 trimer is required for delivering charged tRNAMet to the ribosome in a reaction requiring the hydrolysis of GTP to GDP (Sokabe et al., 2012). The phosphorylation of *eif2-alpha* on its 49th serine residue (Nukazuka et al., 2008) prevents replenishing GDP with GTP and thus reduces translation initiation by preventing delivery of tRNAMet. The phosphorylation of the mammalian ortholog of *eif-2alpha* occurs on serine 51 in response to a variety of stresses (Taniuchi et al., 2016) including: osmotic stress, ultraviolet light, cold shock, oxidative stress, heat shock, anoxia, and serum starvation.

In *C. elegans,* the phosphorylation of *eif-2alpha* has shown to increase in response to ER stress (Howard et al., 2016; Richardson et al., 2011), osmotic stress (Lee and Strange, 2012), and uncharged tRNAs (Rousakis et al., 2013). This is thought to promote survival by reducing translation in a way that also promotes longevity. Using CRISPR-cas9 genome engineering (Paix et al., 2015) we replaced serine 49 in *eif-2alpha* with alanine, which cannot be phosphorylated. The *C. elegans* line ANR165 harbors this engineered allele in the wild-type N2 background.

The phosphorylation status of *eif-2alpha* in response to hypertonicity, heat, and oxidative stress was probed using an antibody specific to its phosphorylated form. Wild type N2 worms showed an increased in phosphorylated *eif2-alpha* in response to all stresses tested (Figure 1A, dotted box outlines P-*eif2-alpha* bands). Total protein is shown as a loading control. In comparison, phosphorylated *eif-2alpha* was not detectable in ANR165 treated with any of the stresses. This result confirms that serine 49 is the site of phosphorylation in *eif-2alpha* in response to salt stress and is a novel finding for heat and oxidative stress in *C. elegans*.

In addition, *eif2-alpha* S49A mutants were viable, demonstrating that loss of *eif-2alpha* serine 49 phosphorylation is non-lethal. Furthermore, time to develop from egg to egg laying adult was significantly (Wilcoxon rank sum test, p < 0.01, n=15) shortened in *eif2-alpha* S49A mutants (Figure 1B) by a mean of 4 hours compared to N2. This result suggests that the phosphorylation status of *eif2-alpha* serves a role in development which may be related to its effect on translation. A role for *eif2-alpha* in development is not surprising as mutants expressing a phosphomemetic version of it in the ASI neurons were impaired in growth and development (Kulalert et al., 2017).

## Methods

*C. elegans* strains were maintained at 20°C on NMG plates spotted with OP50 as a food source. Hypertonic stress was induced by placing worms on NGM plates containing 200 mM sodium chloride for 3 h. Heat stress was induced by placing worms at 35°C for 1 h. Oxidative stress was induced by placing worms on NMG plates containing 120 µM juglone for 3h. All stresses were performed in the presence of OP50 on day 1 adult worms synchronized by timed egg lays. 10 µg of total protein as determined from DC protein assay (Bio-Rad) from each sample was separated on 4-20% mini-Protean TGX stain-free gels (Bio-Rad). The resulting gels were exposed to UV for 5 m to allow fluorescent detection of total protein present.

## Reagents

Strains: N2, ANR165 : *eif-2alpha*(*rog3*[S49A]) Antibody: Phospho-eif2α (Ser51) #9721, Cell Signaling Technology
