# Erratum: Loss of *eif-2alpha* phosphorylation on S49 (mammalian S51) associated with the integrated stress response hastens development in *C. elegans​*

**DOI:** 10.17912/micropub.biology.000229

**Published:** 2020-02-27

## Description

Rollins, J; Lind, N; Rogers, AN (2017). Loss of *eif-2alpha* phosphorylation on S49 (mammalian S51) associated with the integrated stress response hastens development in *C. elegans​*. microPublication Biology. 10.17912/W2BM1S published online Nov 22, 2017 @ 21:16; corrected after publication Feb 27, 2020.

In the version of the microPublication originally published online, the name of the first author was incorrectly spelled. The correct name is **Jarod Rollins.** This mistake has been corrected online.

